# Veterinary Drug Shops as Main Sources of Supply and Advice on Antimicrobials for Animal Use in the Mekong Delta of Vietnam

**DOI:** 10.3390/antibiotics8040195

**Published:** 2019-10-25

**Authors:** Doan Hoang Phu, Vu Thi Quynh Giao, Dinh Bao Truong, Nguyen Van Cuong, Bach Tuan Kiet, Vo Be Hien, Guy Thwaites, Jonathan Rushton, Juan Carrique-Mas

**Affiliations:** 1 Oxford University Clinical Research Unit, HCMC, Vietnam; giaovtq@oucru.org (V.T.Q.G.); baotd@oucru.org (D.B.T.); cuongnv@oucru.org (N.V.C.); gthwaites@oucru.org (G.T.); jCarrique-Mas@oucru.org (J.C.-M.); 2 Faculty of Animal Science and Veterinary Medicine, Nong Lam University, HCMC, Vietnam; 3 Sub Department of Animal Health, Cao Lanh city, Dong Thap, Vietnam; bachkiettydt1@gmail.com (B.T.K.); hienthuydt@gmail.com (V.B.H.); 4 Centre for Tropical Medicine and Global Health, Oxford University, Oxford OX3 7FZ, UK; 5 Institute of Infection and Global Health, University of Liverpool, Liverpool L69 7BE, UK; J.Rushton@liverpool.ac.uk

**Keywords:** veterinary drug shop, farmers, antimicrobial sales, Vietnam

## Abstract

In the Mekong Delta of Vietnam, small-scale poultry farmers use large amounts of antimicrobials to raise their flocks, and veterinary drug shops owners and their staff are a key source of advice to farmers on antimicrobial use (AMU). We described the network of veterinary drug shops (*n* = 93) in two districts within Dong Thap province (Mekong Delta). We also interviewed a randomly selected sample of chicken farmers (*n* = 96) and described their linkages with veterinary drug shops. Antimicrobials represented 15.0% [inter quartile range (IQR) 6.0–25.0] of the shops’ income. Fifty-seven percent shop owners had been/were affiliated to the veterinary authority, 57% provided diagnostic services. The median number of drug shops supplying antimicrobials to each farm during one production cycle was 2 [IQR 1–2]. Visited shops were located within a median distance of 3.96 km [IQR 1.98–5.85] to farms. Drug shops owned by persons affiliated to the veterinary authority that did not provide diagnostic services had a higher fraction of their income consisting of antimicrobial sales (*β* = 1.913; *p* < 0.001). These results suggest that interventions targeting veterinary drug shop owners and their staff aiming at improving their knowledge base on livestock/poultry diseases and their diagnosis may contribute to reducing overall levels of AMU in the area.

## 1. Introduction

The indiscriminate use of antimicrobials in animal production is now recognised as a major driver of antimicrobial resistance (AMR) worldwide [1,2]. Approximately 80% of total antimicrobial usage (AMU) is thought to be aimed at animal production [3]. In the European Union, where data on AMU in humans and animals are regularly collated, AMU in animals represented 70.2% of a total of 12,720 tonnes used in 2014 [4]. Antimicrobials are used in animal production to treat and prevent disease, as well as for growth promotion in many countries [5,6]. In the Mekong Delta of Vietnam, veterinary drug shops are the main source of antimicrobials to chicken farmers [7].

A recent study showed that the density of veterinary drug shops at commune level was positively correlated with AMU in chicken flocks in this area [8]. In Vietnam, in 2015 there were ~10,400 licenced veterinary drug shops (average 150–200 per province). There are approximately ~5000 licensed veterinary products on the market containing more than 70 antimicrobial ingredients [9]. The most significant recent development in the country is the launch (2017) of the National Action Plan (NAP) for the management of AMU and control of AMR in livestock production and aquaculture 2017–2020 [10]. The Vietnamese NAP is aligned with the Food and Agriculture Organisation Action Plan on AMR 2016–2020 [11] and includes key activities to support awareness, surveillance, governance and good AMU practices. However, the Vietnamese NAP does not specifically focus on the network of veterinary drug shops. As in many other low- and middle-income countries (LMICs) antimicrobials are sold ‘over the counter’ without a prescription [12].

High levels of AMU have been reported both in chicken and pig production in the Mekong Delta of Vietnam [13,14,15]. This behaviour is partly driven by the prevailing farming conditions that lead to a high incidence of disease and mortality [8]. In addition, farmers often use antimicrobials prophylactically as a replacement for good farming practices [16]. In the Mekong Delta of Vietnam, there are three to six veterinary drug shops per commune (~32 km^2^), compared with only one or two government veterinarian/s or commune animal health worker/s. Farmers have more regular access to local veterinary drug shops than contact with any other animal health advisors [16]. It has been suggested that pharmacy owners should play a central role in antimicrobial stewardship [17,18]. Therefore, owners of veterinary drug shops and their staff are likely to play an important role in advising farmers on issues related to animal health, including AMU. However, it is of concern that owners and staff of these shops may have vested interests in the sale of antimicrobials. Here, the aims were: (1) To characterise and map out the veterinary drug shop network in the study area; (2) and to investigate linkages between veterinary drug shops with 96 randomly selected chicken farmers in a selected area of the Mekong Delta of Vietnam

## 2. Results

### 2.1. Characteristics of Veterinary Drug Shops

Of the 138 registered drug shops, 45 (32.6%) exclusively sold products for aquaculture. The owners of the remaining 93 veterinary drug shops (i.e., those targeting terrestrial animals) (50 in Cao Lanh, 43 in Thap Muoi) were interviewed. Demographic information and business activities of these shops are described in Table 1. Most (66.7%) owners were male and of a median age of 40 [IQR 36–51] years-old. The majority (59.1%) of veterinary shop owners had a vocational (animal science) qualification obtained in a technical college, whilst the remainder had a degree in veterinary medicine. One owner had a post-graduate (Master’s) degree. Farmers were the main customer of these establishments (median 100% [IQR 90–100]), followed by animal health workers (mentioned by 36.6% veterinary drug shop owners). The median number of staff members (including the owner) working in each shop was 2 [IQR 1–2], the respective values were 2 [IQR 2–2] and 2 [IQR 1–2] for Cao Lanh and Thap Muoi. In term of staffing capacity, the median value was 12 person-days [IQR 7–14] per week, 14 [IQR 9–14] for Cao Lanh and 10 [7,8,9,10,11,12,13,14] for Thap Muoi drug shops. There were 5 (5.4%) shops staffed by personnel adding a total of 22.5–28.0 days/week. A total of 53 (57%) shop owners had links with the local veterinary authority (Sub-Department of Animal Health of Dong Thap, SDAH-DT) (50% in Cao Lanh and 65% in Thap Muoi). Of those, 6 (6.5%) shop owners were currently working at SDAH-DT. A total of 68.8% of shops provided loan services to farmers for specific goods, consisting of commercial feed (60.2% shops) and veterinary medicines (34.4%). Of the total income of antimicrobial sales, products for pig farming represented 30% [IQR 15–60%] income, followed by ducks (20% [IQR 13–40%]), chickens (15% [IQR 10–25]), aquatic species (0% [IQR 0–3]) and other (cows, goats) (2 [IQR 0–10]) respectively. Diagnostic services (including post-mortem necropsy) were available in 57% drug shops (76.0% in Cao Lanh and 34.8% in Thap Muoi). 

**Figure 1:**
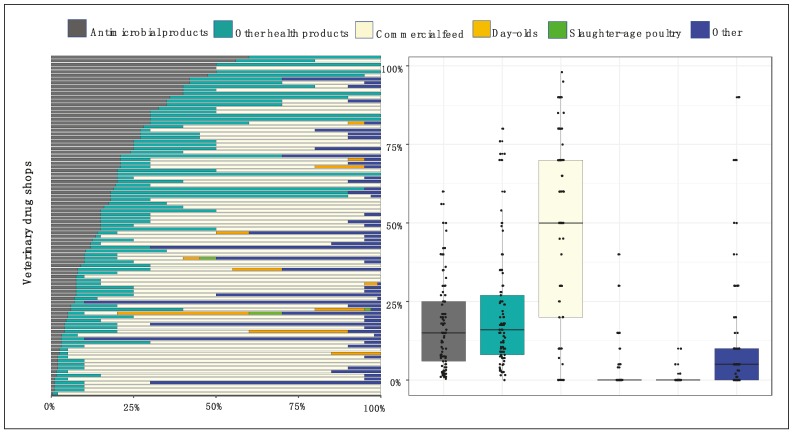
Types of commodities and services provided by veterinary drug shops in the study area. Each colour represents a type of commodity/service, including antimicrobials, non-antimicrobial health-supporting products, commercial feed, day-old-chicks, collection slaughter-age chickens, and ‘Other’ (services and other equipment: Drinkers, feeders, rice husks, etc.).

The types of commodities and services offered by veterinary drug shop are displayed in Figure 1. A total of 85% shops retailed commercial feed, representing a median of 50.0% [IQR 20.0–70.0] of total income across shops. All shops dispensed antimicrobial products and other health-related products. Antimicrobial sales represented a median of 15.0% [IQR 6.0–25.0] of income. Non-antimicrobial drugs health-related products represented a median income of 16.0% [IQR 8.0–27.0] of income). A total of 11.8% and 3.2% veterinary drug shops respectively offered services, such as selling day-old chicks and collecting slaughtered-age chickens.

### 2.2. Mapping of Veterinary Drug Shops and Livestock Populations

**Figure 2:**
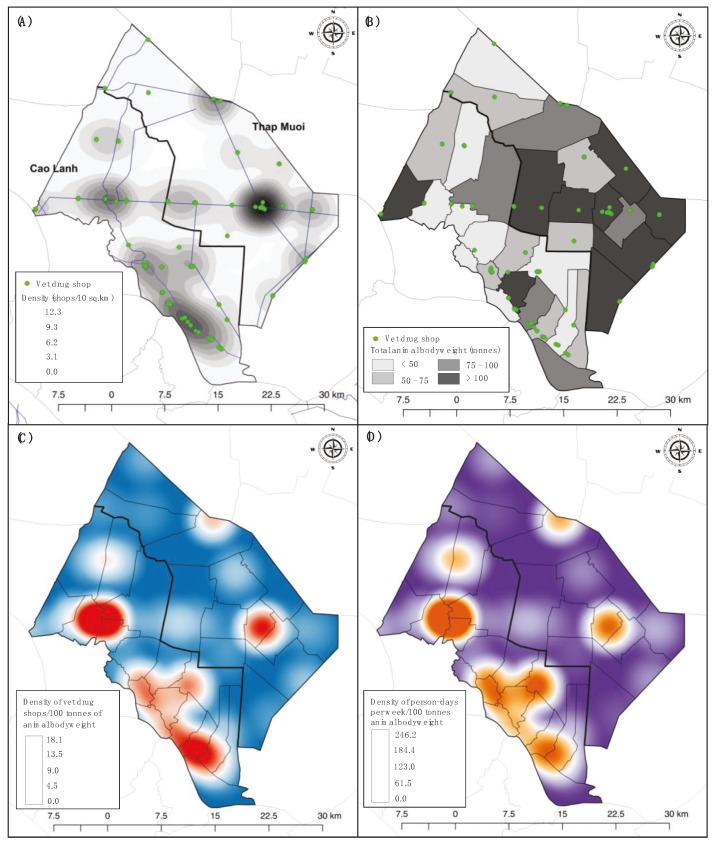
Geographical distribution of veterinary drug shop and livestock density at commune level in the study area. (**A**) Geographical distribution and kernel density of veterinary drug shops within an area of 10 km^2^; (**B**) density of animal populations by commune expressed as animal bodyweight, and location of veterinary drug shops; (**C**) density heat map displaying the number of veterinary drug shops per 100 tonnes of animal bodyweight; (**D**) density heat map of staffing capacity (person-days/week) per 100 tonnes of animal bodyweight.

The overall mean density of drug shops in the study area was 1.76 (SD ±1.94) per 10 km^2^ (1.84/10 km^2^ in Cao Lanh; 1.48/10 km^2^ in Thap Muoi). Drug shops were unevenly distributed across the geographical space, with 5 clusters with a density of >5 shops/10 km^2^ accounting for 47% of all shops (Figure 2). Overall, there were 745 ducks, 167 chickens, 36 pigs, and <8 of each other species (Muscovy ducks, goats, geese, cows etc.) per km^2^ in the two study districts, representing an average of 2.5 tonnes of animal bodyweight per km^2^ (2.4–2.6 in each study district).

Overall there were 3.8 shops (total 46.3 person-days) per 100 tonnes of animal bodyweight, 4.5 in Cao Lanh (57.2 person days), 3.2 (37.3 person-days) in Thap Muoi. These values were equivalent to 1 veterinary drug shop per 26.3 tonnes (~32,875 chickens/ducks or 669 pigs), and 1 person-day/week per 2.2 tonnes (~2750 chickens/ducks or 56 pigs) (Table 2).

### 2.3. Correlation between Number of Veterinary Drug Shops and Livestock Population

There were no significant correlations between the number of veterinary drug shops and the total animal bodyweight, as well as each of the three major species (duck, chicken and pig) at commune level. The Spearman’s rank correlation coefficients were of 0.20, 0.19, 0.08 and 0.17 respectively, (all *p* > 0.280). Similarly, the animal population of total bodyweight and three species of duck, chicken and pig were also not significantly correlated with the staffing capacity per commune (all *p* > 0.520), the correlation coefficients were 0.07, 0.12, 0.05 and 0.06 respectively. (Table S2, Figure S1).

### 2.4. Linkages between Veterinary Drug Shops and Chicken Farms

**Figure 3:**
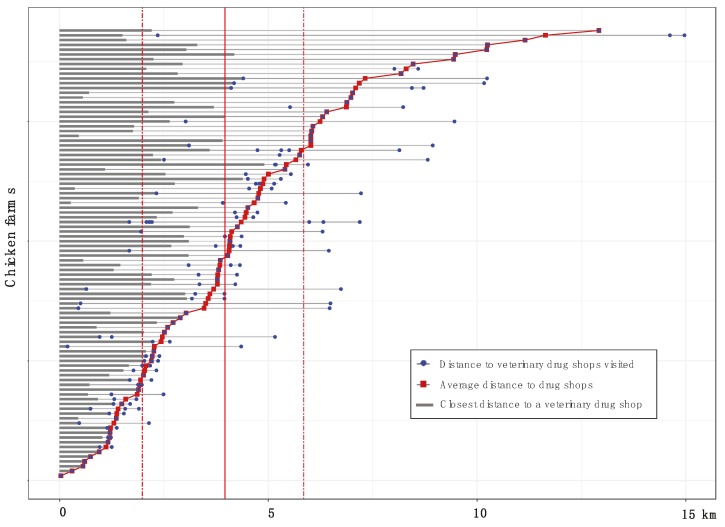
The geographical distance between farms (*n* = 94), their closest veterinary drug shop, and the visited veterinary drug shops from which farmers purchased antimicrobials. Data from 2 farms were excluded, since farmers visited other districts to buy antimicrobials. Solid red line: The median distance between farms and visited shops (3.96 km); Dashed red lines: Inter quartile range [1.98–5.85].

A total of 96 chicken farmers were interviewed. The median number of veterinary drug shops visited by each farmer to purchase antimicrobials was 2 [IQR 1–2]. Farmers in Cao Lanh visited more veterinary drug shops than those in Thap Muoi (median 2 [IQR 2–2] vs. 1 [IQR 1–2] respectively) (Fisher’s Exact Test, *p* < 0.000). Three farmers (3.1%) (2 in Cao Lanh, 1 in Thap Muoi) had visited >3 shops to buy antimicrobials, and 1 bought antimicrobial from 8 different veterinary drug shops. Two farmers had travelled outside their district for purchasing antimicrobials (Table S3). The distance between farms and the shops where farmers had purchased antimicrobials ranged from 0.03 to 14.97 km (median 3.96, [IQR 1.98–5.85]; 2.33 [IQR 1.43–4.36] for Cao Lanh, 5.15 [IQR 3.80–8.25] for Thap Muoi). Farms were located at a median distance of 1.92 km [IQR 0.96–2.76] from their closest veterinary drug shop (1.25 [IQR 0.73–2.06] for Cao Lanh, 2.53 [IQR 1.51–3.08] for Thap Muoi) (Figure 3). 

**Figure 4:**
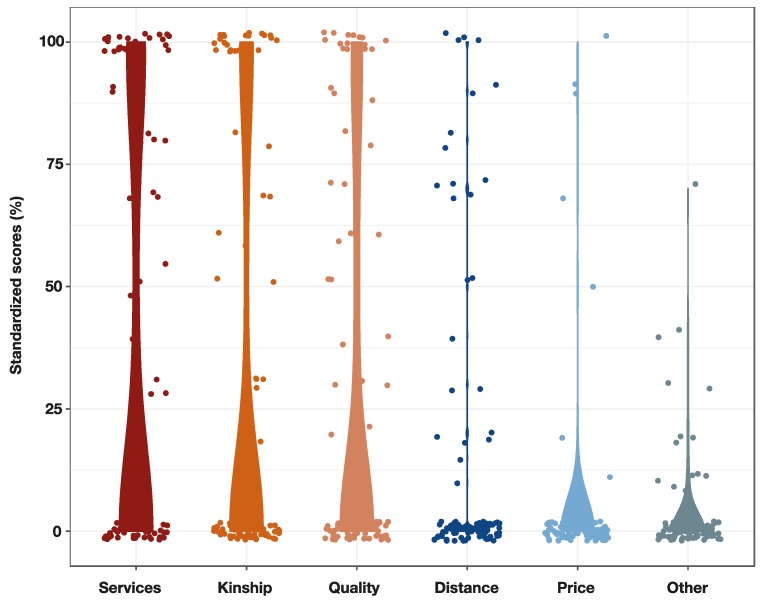
Standardised scores for the reasons stated by farmers for their choice of veterinary drug shops.

The reasons given by farmers to justify their choice of specific veterinary drug shops were, in decreasing order: (1) Animal health services (including advice on husbandry, diagnostic support, including post-mortem, vaccination support, sales of day-old-chicks) (mentioned by 47.9% farmers) (standardised score 38.5%); (2) Kinship (a relative or friend), mentioned by 37.5% farmers (score 31.7%); (3) Quality of products retailed (including the perception of being effective) (mentioned by 37.5% farmers (score 27.6%); (4) Distance from farm (26% farmers) (score 14.7%); and (5) price (7.2% farmers) (score 4.8%). Other, less commonly mentioned reasons (mentioned by 14.6% farmers in total) were: Knowledge, experience of qualification of the owner, availability of special products (drugs used in the brooding period) (scores 4.1) (Figure 4).

### 2.5. Risk Factor Analyses

Factors associated with a higher proportion of their income associated with antimicrobial sales in univariable models (*p* < 0.2) were: (1) Owner ≥ 40 years-old; (2) District of Thap Muoi; (3) Person-days per week (protective) (log), (4) Affiliation to government veterinary authority; and (5) Diagnostic services available. The latter two variables (Affiliation to government veterinary authority and Diagnostic services available) were combined into a new variable with four levels. In the final multivariable model, the variable ‘District’, ‘Age’, and ‘Person-days’ became non-significant, since they were confounded by affiliation to the veterinary authority. The highest risk corresponded to shops owned by veterinarians affiliated to the veterinary authority that did not provide diagnostic services (Table 3).

## 3. Discussion

To our knowledge, this is the first study describing veterinary drug shops and their linkages with farmers in a low- and middle-income country. Our results indicate a poor spatial correlation between veterinary drug shops and animal populations at commune level. This is consistent with our observation that farmers often purchase antimicrobials from shops for reasons other than geographical proximity, preferring to travel to longer distances. In contrast, the provision of services (diagnostics, vaccination support, advice on flock health) were major factors influencing the farmers’ choice/s of veterinary drug shop. 

According to the farmers’ opinions, antimicrobial retail prices had little impact on their specific choice of veterinary drug shop. This is consistent with a previous study conducted in the area, where poultry farmers stated that they would be willing to accept a three to four-fold hike in prices without altering their AMU behaviour [16]. It has been shown that antimicrobials intended for veterinary use are extremely affordable in the region (average of 0.56 cents of a USD per kilogram treated) and represent only a small fraction of overall chicken production costs [12]. We found that, despite high levels of AMU by chicken flocks in the area [15], antimicrobial sales represented a relatively small fraction of the total income of veterinary drug shops. There were, however, the large difference across establishments.

We found interesting differences in antimicrobial sales depending on the geographical location and the profile of the shop owner. Shops in Thap Muoi district obtained a higher fraction of their income from antimicrobial sales. However, this was explained by a higher fraction of drug shops in this district that did not offer diagnostic services. Interestingly, the provision of diagnostic services was not linked to the shop being owned by a fully qualified veterinarian (data not shown). Antimicrobials were more likely sold in establishments where diagnostic support services, even basic (i.e., post-mortem), were not available. This is consistent with studies in human medicine showing that uncertainty of diagnosis or the absence of diagnostic facilities is factors leading to an excessive prescription of antimicrobials [19,20]. 

The higher density of pharmacies in Cao Lanh district is likely to result in competition among shop owners and be reflected in more likely availability of diagnostic services in their shops. Despite differences observed in antimicrobial sales and staff capacity in shops in these two districts, a previous study identified larger overall levels of AMU among chicken farmers in this district [8]. This was probably explained by a larger number of veterinary drug shops accessed by farmers in this district. 

We mapped out veterinary drug shops and related these to animal bodymass. A previous study conducted in one district within Ho Chi Minh City estimated that there were 301 drug shops for a resident human population of 396,175 people [21]. Assuming an average bodyweight of 50 kilograms per person, we calculate that one pharmacy supplied to a total of 65.8 tonnes of human bodyweight. In contrast, in our study, there was one veterinary drug shop for 26.3 tonnes of animal bodyweight (i.e., 2.5 times higher than human drug shops). 

Our study had a number of limitations: We only interviewed drug shop owners, even though other persons for which we did not gather information often staffed these shops. Also, there were a number of veterinary drug shops falling outside the district boundaries. This was more likely for farms located close to the edges of the district (data not shown). This may have resulted in an underestimation of the distances between farms and their chosen drug shops. We focused on small- scale chicken farms since small-scale farming is the most common type of farming system in the Mekong Delta and elsewhere in Southeast Asia. The chosen farms had already been enrolled as part of a large field-based project (www.viparc.org), and previous data indicated exceptionally high levels of AMU in these systems. We believe that, to a certain extent, our findings can be extrapolated to small farms raising other poultry species and pigs in the region.

## 4. Materials and Methods

### 4.1. Study Area, Populations and Veterinary Drug Shops

The study was conducted in two districts (Cao Lanh and Thap Muoi) within Dong Thap province (Mekong Delta of Vietnam) in October 2018. These two districts had a combined area of 982.6 km^2^, representing 27% of the whole province, and have a combined population of 313,445 people (population density 319 people/km^2^). Rice and fruit crops, as well as raising livestock (pigs, cattle, goats) and poultry (ducks, chickens and Muscovy ducks) are the main economic activities in this rural area. Data on animal populations by commune (an administrative sub-division within the district) were provided by the Sub-Department of Animal Health of Dong Thap (SDAH-DT) (official census, 2017). In Cao Lanh and Thap Muoi districts, there were a combined population of 732,337 ducks, 163,572 chickens, 35,647 pigs, 7843 Muscovy ducks, 2934 cows, 1160 goats, and 784 geese. A total of 138 active veterinary drug shops were registered in these two districts. 

### 4.2. Correlation between Veterinary Drug Shop and Livestock Population

Data on the total number of animals (ducks, Muscovy ducks, chickens, pigs, geese, bovines, goats) in each commune were converted into animal bodyweight based on 50% of the average weight of slaughtered animals in Mekong region [22]: Duck, chicken (1.6 kg), goat (44.4 kg), pig (78.6 kg), cow (200 kg). The Muscovy duck and goose slaughter weights were estimated in 3.2 kg. We calculated the Spearman’s rank correlation coefficient between the number of veterinary drug shops and animal bodyweight at commune level. Detailed on animal population by species at commune level are provided in Table S2.

### 4.3. Mapping of Veterinary Drug Shops and Livestock Density

The location coordinates of all veterinary drug shops and chicken farms in the two study districts were obtained. These were plotted using Quantum GIS (QGIS), version 2.18.15 (QGIS Development Team) based on DIVA-GIS boundary data (https://www.diva-gis.org). A kernel density algorithm was used to create density heat maps of veterinary drug shop within a radius of 5 kilometers [23]. Likewise, the ratio of veterinary drug shops and person-days per week (the sum of working days of all staff including the shop owner per week) to total tonnes of animal bodyweight per commune was plotted using a kernel density.

### 4.4. Survey of Veterinary Drug Shops and Chicken Farmers

Veterinary drug shop owners in Cao Lanh and Thap Muoi district were interviewed using structured questionnaires. Information on demographic characteristics of the shop owners (i.e., age, gender, educational status) and other shop-related variables (district, number of years in business, type/s of customer, opening times, staffing capacity, types of products sold, diagnostic services, loan service of feed, health products and sales by species) were collected. In addition, we interviewed small-scale chicken farmers (raising between 100 and 2000 chickens) that had previously been randomly selected for a longitudinal study [24]. Farmers were asked to list the veterinary drug shops from where they purchased veterinary drugs over their latest flock production cycle. Farmers were asked to list and rank the reasons behind their choice of each drug shop, adding up to 100%. We calculated a standardised score for each reason by multiplying each of these ranks by the share of expenditure on each veterinary drug shop. The distances between veterinary drug shops and chicken farms were determined using the Distance Matrix Tool on QGIS.

### 4.5. Risk Factor Analysis

Risk factor analyses for the outcome variable ‘proportion of business income consisting of antimicrobial sales (square root transformed) were carried out by linear regression. The variables investigated were: (1) Owner’s gender; (2) Owner’s age (as determined with a cut-off of median value of 40 years old); (3) Qualification of owner (vocational/bachelor or higher); (4) District; (5) Numbers of years in business (log); (6) Staffing capacity (person-days per week) (log); (7) Affiliation to veterinary authority (previous or current); (8) Diagnostic services available (including post-mortem); (9) Loan services available; (10) Kernel density of veterinary drug shop (log). A multivariable model was built using a step-wise forward approach to select the final model. Univariable models were screened, and those with a *p* < 0.20 were kept as a candidate for multivariable models. All statistical analyses were done using R (http://www.r-project.org).

## 5. Conclusions

Our findings suggest that improving the knowledge base of veterinary drug shops owners and their staff on animal diseases and diagnostics may contribute to reducing excessive dispensation of antimicrobials, whilst improving their awareness on the consequences of antimicrobial misuse. This should be coupled with more stringent licencing requirements and training certificates to owners of these shops, as well as any staff operating them.

## Figures and Tables

**Table 1:** Descriptive characteristics of veterinary drug shops. The breakdown of total sample size into a fraction of antimicrobial sales is based on a median of 15% of antimicrobial sales.

Characteristics	All (*n* = 93)	<15% Antimicrobial Sales (*n* = 45)	≥15% Antimicrobial Sales (*n* = 48)
Owner’s gender (%)			
*Male*	62 (66.7%)	31	31
*Female*	31 (33.3%)	14	17
Owner’s age in year (median [IQR])	40 [36–51]	39 [36–48]	44 [38–52]
Education status (%*)*			
*Master’s degree*	1 (1.1)	1	0
*Degree in Vet Medicine/Animal husbandry*	37 (39.8)	17	20
*Vocational*	55 (59.1)	27	28
District (%)			
*Cao Lanh*	50 (53.8)	29	21
*Thap Muoi*	43 (46.2)	16	27
Number of years in business (median [IQR])	13 [6–18]	12 [6–16]	14 [7–12]
Percent of customer by type (median [IQR])			
*Farmer*	100 [90–100]	100 [97–100]	100 [88–100]
*Other vet shops*	0 [0–0]	0 [0–0]	0 [0–0]
*Animal health worker*	0 [0–10]	0 [0–2]	0 [0–2]
Staffing (median [IQR])			
*No. staff*	2 [1–2]	2 [2–2]	2 [1–2]
*Person-days per week*	12 [7–14]	14 [9–14]	11 [7–14]
Affiliation (%)			
*Veterinary authority (current)*	6 (6.5%)	2	4
*Veterinary authority (previous)*	47 (50.5%)	19	28
*No veterinary authority affiliation*	40 (43.0%)	24	16
Diagnostic service available (%)			
*Yes*	53 (57.0%)	32	21
*No*	40 (43.0%)	13	27
Loan service (%)			
*Any product (feed or health products)*	64 (68.8)	32	32
*Feed*	56 (60.2)	31	25
*Health products*	32 (34.4)	14	18
Percent of antimicrobial sales by species (median/[IQR])			
*Pig*	30 [15–60]	30 [15–70]	30 [18–60]
*Duck*	20 [13–40]	25 [10–50]	20 [15–36]
*Chicken*	15 [10–25]	10 [10–20]	15 [10–25]
*Aquatic animals*	0 [0–3]	0 [0–3]	0 [0–5]
*Other*	2 [0–10]	4 [0–10]	1 [0–10]

**Table 2:** Veterinary drug shops and density of animal population by commune and district.

Commune	Area (km^2)^	Vet Drug Shops	Person- Days per Week (Sum)	Person-Days per Week (Average)	Total Tonnes Animal Bodyweight	Tonnes Bodymass per km^2^	No. of Drug Shops per 100 tonnes	Person-Days per Week per 100 tonnes
Cao Lanh District	464.7	50	635.0	12.7	1109.7	2.4	4.5	57.2
An Binh	8.2	0	0.0	0.0	28.9	3.5	0.0	0.0
Ba Sao	62.7	2	22.5	11.3	77.9	1.2	2.6	28.9
Binh Hanh Tay	14.4	6	63.0	10.5	60.7	4.2	9.9	103.9
Binh Hang Trung	19.9	4	31.5	7.9	95.6	4.8	4.2	32.9
Binh Thanh	27.6	0	0.0	0.0	75.5	2.7	0.0	0.0
Gao Giong	51.4	3	43.0	14.3	59.8	1.2	5.0	72.0
My Hiep	22.5	2	21.0	10.5	72.2	3.2	2.8	29.1
My Hoi	15.9	4	46.0	11.5	105.4	6.6	3.8	43.7
My Long	21.0	3	42.0	14.0	42.7	2.0	7.0	98.5
My Tho	23.9	1	7.0	7.0	66.8	2.8	1.5	10.5
My Tho town	8.2	5	75.0	15.0	59.2	7.2	8.4	126.7
My Xuong	9.9	2	30.5	15.3	39.5	4.0	5.1	77.1
Nhi My	26.0	1	14.0	14.0	49.2	1.9	2.0	28.4
Phong My	28.2	3	35.0	11.7	103.2	3.7	2.9	33.9
Phuong Thinh	44.3	2	38.0	19.0	45.1	1.0	4.4	84.2
Phuong Tra	17.5	6	81.5	13.4	33.1	1.9	18.1	246.2
Tan Hoi Trung	40.7	4	70.0	17.5	46.2	1.1	8.7	151.6
Tan Nghia	22.4	2	15.0	7.5	48.7	2.2	4.1	30.8
Thap Muoi District	517.7	43	502.0	11.7	1346.9	2.6	3.2	37.3
Doc Binh Kieu	32.6	4	73.5	18.8	216.0	6.6	1.9	34.0
Hung Thanh	49.6	1	7.0	7.0	74.0	1.5	1.4	9.5
Lang Bien	23.3	1	7.0	7.0	73.4	3.1	1.4	9.5
My An	18.6	1	9.5	9.5	97.4	5.2	1.0	9.8
My An town	17.1	12	149.5	12.5	107.9	6.3	11.1	138.6
My Dong	25.2	1	7.0	7.0	108.7	4.3	0.9	6.4
My Hoa	36.7	2	15.0	7.5	63.4	1.7	3.2	23.7
My Quy	61.3	6	63.0	10.5	112.0	1.8	5.4	56.2
Phu Dien	45.4	4	40.5	10.1	143.8	3.2	2.8	28.2
Tan Kieu	42.4	2	16.0	8.0	100.1	2.4	2.0	16.0
Thanh Loi	47.8	1	14.0	14.0	45.9	1.0	2.2	30.5
Thanh My	44.7	2	14.0	7.0	127.2	2.8	1.6	11.0
Truong Xuan	73.0	6	86.0	14.3	77.1	1.1	7.8	111.5
Whole study area	982.4	93	1137	12.2	2456.6	2.5	3.8	46.3

**Table 3:** Linear models that investigate factors associated with a higher share of income consisting of antimicrobial sales.

	Univariable			Multivariable *		
	*β*	95% CI	*p*-Value	*β*	95% CI	*p*-Value
Owner’s gender (baseline = Male)						
Female	0.011	−0.77–0.79	0.976			
Owner’s age (baseline <40 years old)						
≥40 years old	0.647	−0.07–1.37	0.079	0.156	−0.57–0.88	0.672
Education status (baseline = Vocational)						
Bachelor or higher	0.341	−0.40–1.08	0.366			
District (baseline = Cao Lanh)						
Thap Muoi	0.993	0.28–1.70	0.006	0.462	−0.31–1.24	0.241
No. of years in business (log)	0.207	−0.18–0.59	0.296			
Person-days per week (log)	−0.910	−1.81–0.00	0.049	−0.726	−1.62–0.16	0.110
Affiliation to veterinary government authority (baseline = No)						
Yes	0.773	0.04–1.50	0.037			
Diagnostic service (baseline = Yes)						
No	0.989	0.27–1.70	0.007			
Affiliation of shop owner and diagnostic service (baseline = No affiliation to veterinary government authority, diagnostic service)						
Affiliation to veterinary government authority, diagnostic service	0.656	−0.28–1.59	0.167	0.565	−0.38–1.51	0.239
Affiliation to veterinary government authority, no diagnostic service	1.913	0.88–2.94	<0.001	1.497	0.32–2.66	0.012
No affiliation to veterinary government authority, no diagnostic service	0.801	−0.25–1.85	0.135	0.692	−0.44–1.82	0.229
Loan service of any product (baseline = Yes)						
No	0.454	−0.33–1.24	0.257			
Kernel density of shops (log)	−0.087	−0.67–0.50	0.769			

* Intercept = 4.578; SE = 1.156.

## Supplementary Materials

The following are available online at https://www.mdpi.com/2079-6382/8/4/195/s1, Figure S1: Correlation between animal population and number of veterinary drug shops/ total person-day per week at commune level, Table S1: Data on survey of veterinary drug shop, Table S2: Total animal population in study area, Table S3: Geographical coordinates and distance between veterinary drug shops and chicken farms, Table S4. Standardised scores of reasons for choosing a veterinary drug shop by chicken farmers.

## References

[1] Ayukekbong J.A., Ntemgwa M., Atabe A.N. (2017). The threat of antimicrobial resistance in developing countries: Causes and control strategies. Antimicrob. Resist. Infect. Control.

[2] Zellweger R.M., Carrique-Mas J., Limmathurotsakul D., Day N.P.J., Thwaites G.E., Baker S., Ashley E., de Balogh K., on behalf of the Southeast Asia Antimicrobial Resistance Network, Members of the Southeast Asia Antimicrobial Resistance Network (2017). A current perspective on antimicrobial resistance in Southeast Asia. J. Antimicrob. Chemother..

[3] WHO Stop Using Antibiotics in Healthy Animals to Prevent the Spread of Antibiotic Resistance. https://www.who.int/news-room/detail/07-11-2017-stop-using-antibiotics-in-healthy-animals-to-prevent-the-spread-of-antibiotic-resistance.

[4] ECDC/EFSA/EMA First Joint Report on the Integrated Analysis of the Consumption of Antimicrobial Agents and Occurrence of Antimicrobial Resistance in Bacteria from Humans and Food-Producing Animals; Joint Interagency Antimicrobial Consumption and Resistance Analysis (JIACRA) Report; p. 114. https://efsa.onlinelibrary.wiley.com/doi/10.2903/j.efsa.2015.4006.

[5] Roess A., Leibler J.H., Graham J.P., Lowenstein C., Waters W.F. (2016). Animal Husbandry Practices and Perceptions of Zoonotic Infectious Disease Risks Among Livestock Keepers in a Rural Parish of Quito, Ecuador. Am. J. Trop. Med. Hyg..

[6] Van Boeckel T.P., Brower C., Gilbert M., Grenfell B.T., Levin S.A., Robinson T.P., Teillant A., Laxminarayan R. (2015). Global trends in antimicrobial use in food animals. Proc. Natl. Acad. Sci. USA.

[7] Carrique-Mas J.J., Trung N.V., Hoa N.T., Mai H.H., Thanh T.H., Campbell J.I., Wagenaar J.A., Hardon A., Hieu T.Q., Schultsz C. (2015). Antimicrobial Usage in Chicken Production in the Mekong Delta of Vietnam. Zoonoses Public Health.

[8] Carrique-Mas J., Van N.T.B., Cuong N.V., Truong B.D., Kiet B.T., Thanh P.T.H., Lon N.N., Giao V.T.Q., Hien V.B., Padungtod P. (2019). Mortality, disease and associated antimicrobial use in commercial small-scale chicken flocks in the Mekong Delta of Vietnam. Prev. Vet. Med..

[9] Ngo T.T. (2015). Veterinary Medicine Trading according to Veterinary Law in 2015. Master of Law, Vietnam Academy of Social Science: Ha Noi. http://vannghiep.vn/wp-content/uploads/2018/04/Kinh-doanh-thuốc-thú-y-theo-Luật-Thú-y-năm-2015-.pdf.

[10] Summary of the Viet Nam Action Plan for AMU/AMR Reduction in Livestock Sector|FAO in Viet Nam|Food and Agriculture Organization of the United Nations. http://www.fao.org/vietnam/news/detail-events/en/c/451446/.

[11] The FAO Action Plan on Antimicrobial Resistance 2016–2020|Global Forum on Food Security and Nutrition (FSN Forum). http://www.fao.org/fsnforum/resources/fsn-resources/fao-action-plan-antimicrobial-resistance-2016-2020.

[12] Carrique-Mas J., Van Cuong N., Truong B.D., Phu D.H., Phuc T.M., Turner H., Thwaites G., Baker S. (2019). Affordability of antimicrobials for animals and humans in Vietnam: A call to revise pricing policies. Int. J. Antimicrob. Agents.

[13] Cuong N., Padungtod P., Thwaites G., Carrique-Mas J. (2018). Antimicrobial Usage in Animal Production: A Review of the Literature with a Focus on Low- and Middle-Income Countries. Antibiotics.

[14] Nhung N., Cuong N., Thwaites G., Carrique-Mas J. (2016). Antimicrobial Usage and Antimicrobial Resistance in Animal Production in Southeast Asia: A Review. Antibiotics.

[15] Cuong N.V., Phu D.H., Van N.T.B., Dinh Truong B., Kiet B.T., Hien B.V., Thu H.T.V., Choisy M., Padungtod P., Thwaites G. (2019). High-Resolution Monitoring of Antimicrobial Consumption in Vietnamese Small-Scale Chicken Farms Highlights Discrepancies Between Study Metrics. Front. Vet. Sci..

[16] Truong D.B., Doan H.P., Doan Tran V.K., Nguyen V.C., Bach T.K., Rueanghiran C., Binot A., Goutard F.L., Thwaites G., Carrique-Mas J. (2019). Assessment of Drivers of Antimicrobial Usage in Poultry Farms in the Mekong Delta of Vietnam: A Combined Participatory Epidemiology and Q-Sorting Approach. Front. Vet. Sci..

[17] Erickson A.K. (2016). Hospital pharmacists are essential to antimicrobial stewardship. Pharm. Today.

[18] (2010). ASHP Statement on the Pharmacist’s Role in Antimicrobial Stewardship and Infection Prevention and Control. Am. J. Health. Syst. Pharm..

[19] Kotwani A., Wattal C., Katewa S., Joshi P.C., Holloway K. (2010). Factors influencing primary care physicians to prescribe antibiotics in Delhi India. Fam. Pract..

[20] Biedron C., Chopra T. (2013). Issues Surrounding Antibiotic Use in Older Adults. Curr. Transl. Geriatr. Exp. Gerontol. Rep..

[21] Thi Quynh Nhi L., de Alwis R., Khanh Lam P., Nhon Hoa N., Minh Nhan N., Thi Tu Oanh L., Thanh Nam D., Nguyen Ngoc Han B., Thi Thuy Huyen H., Thi Tuyen D. (2018). Quantifying antimicrobial access and usage for paediatric diarrhoeal disease in an urban community setting in Asia. J. Antimicrob. Chemother..

[22] Teufel N., Markemann A., Kaufmann B., Zárate A.V., Otte J. Livestock Production Systems. http://www.fao.org/3/a-bp184e.pdf.

[23] QGIS Heatmap Using Kernel Density Estimation Explained. https://www.geodose.com/2017/11/qgis-heatmap-using-kernel-density.html.

[24] Carrique-Mas J.J., Rushton J. (2017). Integrated Interventions to Tackle Antimicrobial Usage in Animal Production Systems: The ViParc Project in Vietnam. Front. Microbiol..

